# Identification and Characterization of Thermostable Y-Family DNA Polymerases η, ι, κ and Rev1 From a Lower Eukaryote, *Thermomyces lanuginosus*

**DOI:** 10.3389/fmolb.2021.778400

**Published:** 2021-11-03

**Authors:** Alexandra Vaisman, John P. McDonald, Mallory R. Smith, Sender L. Aspelund, Thomas C. Evans, Roger Woodgate

**Affiliations:** ^1^ Laboratory of Genomic Integrity, National Institute of Child Health and Human Development, National Institutes of Health, Bethesda, MD, United States; ^2^ New England Biolabs Incorporated, Ipswich, MA, United States

**Keywords:** thermostable fungi, Y-family DNA polymerases, phylogenetic analysis, translesion DNA synthesis, DNA polymerase eta (polη), DNA polymerase iota (polι), DNA polymerase kappa (polκ), Rev1

## Abstract

Y-family DNA polymerases (pols) consist of six phylogenetically separate subfamilies; two UmuC (polV) branches, DinB (pol IV, Dpo4, polκ), Rad30A/POLH (polη), and Rad30B/POLI (polι) and Rev1. Of these subfamilies, DinB orthologs are found in all three domains of life; eubacteria, archaea, and eukarya. UmuC orthologs are identified only in bacteria, whilst Rev1 and Rad30A/B orthologs are only detected in eukaryotes. Within eukaryotes, a wide array of evolutionary diversity exists. Humans possess all four Y-family pols (pols η, ι, κ, and Rev1), *Schizosaccharomyces pombe* has three Y-family pols (pols η, κ, and Rev1), and *Saccharomyces cerevisiae* only has polη and Rev1. Here, we report the cloning, expression, and biochemical characterization of the four Y-family pols from the lower eukaryotic thermophilic fungi, *Thermomyces lanuginosus*. Apart from the expected increased thermostability of the *T. lanuginosus* Y-family pols, their major biochemical properties are very similar to properties of their human counterparts. In particular, both Rad30B homologs (*T. lanuginosus* and human polɩ) exhibit remarkably low fidelity during DNA synthesis that is template sequence dependent. It was previously hypothesized that higher organisms had acquired this property during eukaryotic evolution, but these observations imply that polι originated earlier than previously known, suggesting a critical cellular function in both lower and higher eukaryotes.

## Introduction

The Y-family DNA polymerases are responsible for copying damaged DNA during DNA replication in a process called translesion synthesis (TLS) (Sale et al., 2012). These enzymes are highly specialized in order to accommodate different structural DNA distortions caused by a wide variety of DNA lesions. The Y-family is divided into six phylogenetically distinct subfamilies: two UmuC (polV) branches; Rad30A/POLH (polη); Rad30B/POLI (polι); and DinB (pol IV, Dpo4, polκ); and Rev1 (Ohmori et al., 2001). Across the different domains of life, Y-family polymerase subfamilies are found in various combinations. For example, UmuC orthologs are only detected in Gram-positive and Gram-negative bacteria, whereas Rev1 and Rad30A/B orthologs are only detected in eukaryotes. The DinB subfamily is the most evolutionarily conserved, having members scattered throughout all three domains of life from unicellular bacteria to humans. However, differences in the distribution of Y-family DNA pols are present within each kingdom. For example, the eukaryote *Saccharomyces cerevisiae* (*S. cerevisiae*) contains neither a *POLK* nor a *POLI* gene. Indeed, it was originally assumed that polι was expressed only in higher eukaryotes. However, next generation whole genome sequencing has revealed that polι orthologs are actually distributed throughout the whole Eukaryota domain. One example is the thermophilic fungus, *Thermomyces lanuginosus* (*T. lanuginosus*) which possesses all four eukaryotic Y-family subfamilies much like humans, in contrast to its fungal relatives, *S. cerevisiae* and *Schizosaccharomyces pombe* (*S. pombe*)*.* Is there logic in such seemingly random distribution of polι? Using phylogenetic analysis and comparing the biochemical characterization of Y-family pols from different species, we hoped to shed some light on this question.

The Y-family DNA pols are classically described as specializing in TLS activity that arises from their capacious active sites that accommodate DNA lesions which would otherwise obstruct the processive confined active sites of A- and B-family replicative pols (Sale et al., 2012). Each Y-family polymerase is tailored to process different lesions, leaving behind unique errors after gaining access to the replication fork (Yang and Gao, 2018). Polymerases belonging to the same subfamily often specialize in targeting the same type of DNA lesions. For example, DinB/polκ orthologs are very adept at bypassing minor groove DNA adducts (*e.g., N^2^*-dG adducts) (Liu et al., 2014; Basu et al., 2017; Jha and Ling, 2018; Stern et al., 2019). Although, the archaeal ortholog, Dpo4, is notably less efficient at bypassing bulky aromatic lesions than its eukaryotic ortholog polκ (Avkin et al., 2004; Ling et al., 2004b; Choi et al., 2006). Along with a common preference for bulky aromatics, DinB pols also share a propensity for template slippage that increases deletion events in the mutation spectra (Kokoska et al., 2002).

Eukaryotic Rad30 orthologs polη and polι are similar in sequence but exhibit very different TLS properties. Human and *S. cerevisiae* polη are exceptionally efficient at bypassing a thymine-thymine cyclobutane pyrimidine dimer (CPD) (Johnson et al., 2000c; Masutani et al., 2000; McCulloch et al., 2004). Although polι can insert nucleotides opposite CPDs, the efficiency is substantially lower than polη-catalyzed TLS (Tissier et al., 2000a; Vaisman et al., 2003). Polι also has a unique feature; it misincorporates dG opposite dT, 3- to 10- fold more frequently than the correct base dA (Johnson et al., 2000b; Tissier et al., 2000b; Zhang et al., 2000c; McIntyre, 2020). These dG:dT misinsertions arise from polι′s remarkably large aliphatic side chains in the finger domain, unlike any other Y-family pols (Kirouac and Ling, 2009; Makarova and Kulbachinskiy, 2012). Despite the extremely low fidelity of polι on a template dT, it is moderately accurate when incorporating opposite other target bases. The highest fidelity is found opposite the template A, where polι inserts the correct base dT with error rate of 10^–4^ (Johnson et al., 2000b; Tissier et al., 2000b; Zhang et al., 2000c).

The unique feature of eukaryotic Rev1 orthologs is their efficiency at bypassing both damaged guanines and abasic sites using a deoxycytidyl transferase mechanism that limits Rev1 to exclusively incorporate dC (Nelson et al., 1996). This is achieved by displacing the DNA lesion from the active site entirely and instead using a protein sidechain (R324 in *S. cerevisiae* and R357 in *H. sapiens*) as the “template” which base pairs solely with dC (Nair et al., 2005, 2011; Weaver et al., 2020).

In this manuscript, we describe the identification, purification and characterization of thermostable eukaryotic orthologs of polη, polι, polκ, and Rev1 from a thermophilic, multicellular fungal species, *T*. *lanuginosus*. Biochemical characterization of TLS DNA pols η, ι, κ, and Rev1 include determination of the enzyme’s fidelity, processivity, thermostability, metal ion requirements, and TLS specificity during bypass of CPDs, abasic sites, and benzo[*a*] pyrene diol epoxide (BPDE) adducts. Our findings serve as basis for comparative analysis of the properties of proteins from different species, thus providing an important insight into the functional evolution of the Y-family polymerases.

## Materials and Methods

### Bacterial Plasmids

Plasmids used in this study are described in Table 1. Where noted, bacteria were grown on LB agar plates containing 20 μg/ml chloramphenicol; 25 μg/ml zeocin; 30 μg/ml kanamycin; 20 μg/ml spectinomycin; or 100 μg/ml ampicillin.

**TABLE 1:** Plasmids used in this study.

Plasmid	Relevant characteristics	Source or reference
pGEM-T	TA PCR cloning vector	Promega
pET22b+	Protein expression vector	EMD Millipore
pJM871	Low copy expression vector	Frank et al. (2012)
pJM574	*T. lanuginosus POLH* in pGEM-T	This study
pJM676	*T. lanuginosus POLK* in pGEM-T	This study
pJM861	*T. lanuginosus REV1* in pGEM-T	This study
pUC57_Ec_CO_Tl iota	*T. lanuginosus POLI* in pUC57	Genscript
pJM596	*T. lanuginosus POLH* expression vector (pET22b)	This study
pJM682	*T. lanuginosus POLK* expression vector (pET22b)	This study
pJM863	*T. lanuginosus REV1* expression vector (pET22b)	This study
pJM966	*T. lanuginosus POLI* expression vector (pJM871)	This study

### Identification and Cloning of Y-Family Orthologs From *T. lanuginosus*

At the onset of this investigation, the complete genomic sequence of *T. lanuginosus* was not yet available. Therefore, in order to identify and clone the various Y-family polymerase orthologs from *T. lanuginosus*, we used several PCR-based procedures. Initially, we employed a degenerate PCR approach by first generating protein homology comparisons for known fungal polη, polι, polκ, and Rev1 proteins. Regions of conserved amino acid stretches within these four polymerase families were identified and degenerate PCR primers were designed based on selected conserved amino acid regions. Purified *T. lanuginosus* genomic DNA was purchased from DSMZ (Leibniz Institute DSMZ-German Collection of Microorganisms and Cell Cultures GmbH) (DSM 10635). PCR and nested-PCR were performed utilizing this *T. lanuginosus* genomic DNA and amplicons of gene segments encoding these four Y-family polymerases were cloned and sequenced. Based on the cloned DNA sequences that encoded regions of the Y-family polymerase genes, gene-specific PCR primers were designed. Additional PCR reactions were performed either using genomic DNA or DNA from a *T. lanuginosus* cDNA library, kindly provided by the Fungal Genomics Project at Concordia University (Adrian Tsang; Director of the Center for Functional and Structural Genomics). PCR techniques employed included RACE PCR (cDNA library), Flanking-sequence PCR (Sorensen et al., 1993) utilizing degenerate biotinylated primers and over-lapping exon PCR (genomic DNA). Full-length genes were amplified with gene-specific primers with the addition of an *Nde*I site at the 5′ end and either a *Bam*HI or *Bgl*II (*POLK*) site at the 3′ end and cloned into pGEM vectors for sequencing. The *POLH*, *POLK* and *REV1* genes were subcloned into the *Nde*I to *Bam*HI sites of pET22b+ for protein expression. An *E. coli* codon optimized version of the *POLI* gene was subsequently synthesized by Genscript (Piscataway, NJ) and cloned into the pJM871 low expression vector (Frank et al., 2012) from *Nde*I to *Bam*HI.

Subsequently, the full *T. lanuginosus* SSBP genomic sequence was published and released (https://mycocosm.jgi.doe.gov/Thelan1/Thelan1.home.html) (McHunu et al., 2013) (GenBank assembly accession GCA_000315315935.1), and we were able to confirm that our clones do indeed encode the *T. lanuginosus* polη, polι, polκ, and Rev1 proteins. The *POLH* gene is encoded on contig00146 (Genbank accession ANHP01000280) from nucleotide 84213–86129. The *POLI* gene is encoded on contig00250 (Genbank accession ANHP01000232) from nucleotide 126087–127864 with two introns. The *POLK* gene is encoded on contig00025 (Genbank accession ANHP01000258) from nucleotide 58089–60067 with one intron. The Rev1 gene is encoded on contig00203 (Genbank accession ANHP01000194) from nucleotide 112065–115607 with one intron. The Joint Genome Institute MycoCosm database designations for theses proteins is as follows: polη – jgi|Thelan1|4409|TLAN_03021-R0; polι – jgi|Thelan1|3308|TLAN_02476-R0; polκ – jgi |Thelan1|3900|TLAN_01802-R0; Rev1 – jgi|Thelan1|3900|TLAN_01802-R0.

### Phylogenetic Tree Construction

**FIGURE 1:**
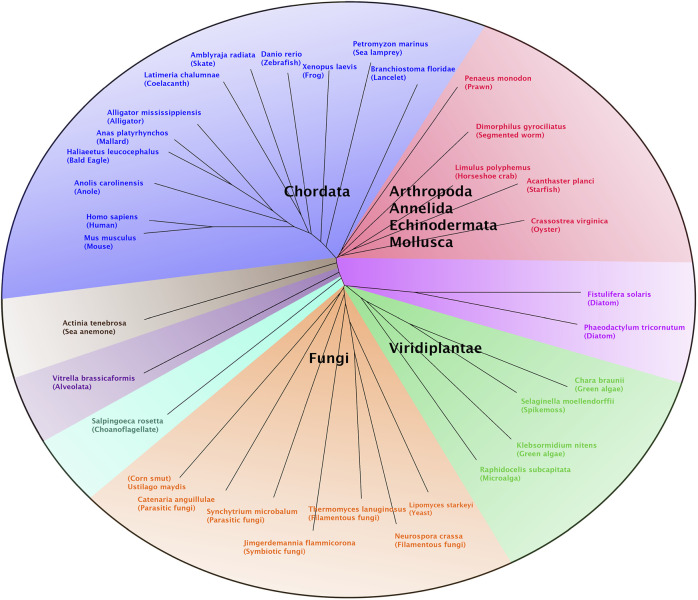
Phylogenetic analysis of polι. A multiple sequence alignment of polι proteins from a broad range of eukaryotic organisms, including *T. lanuginosus* polι and human polι, was performed using the ClustalW algorithm in MacVector (version 15.5.4). This alignment was exported from MacVector as a Nexus file which was then imported into SplitsTree4 (version 4.14.4) to generate an unrooted phylogenetic tree by setting the distance method to BioNJ. Most of the polι proteins cluster into several groups comprised of chordates (blue), lower animals (red), lower plants (green), fungi (orange) and diatoms (pink). Polι protein sequences for each organism were obtained from the following Genbank sequence files: *Acanthaster planci* (XP_022107640), *Actinia tenebrosa* (XP_031566011), *Alligator mississippiensis* (XP_006269716), *Amblyraja radiata* (XP_032889300), *Anas platyrhynchos* (XP_027302772), *Anolis carolinensis* (XP_016850726), *Branchiostoma floridae* (XP_035668094), *Catenaria anguillulae* (ORZ32752), *Chara braunii* (GBG75489), *Crassostrea virginica* (XP_022331447), *Danio rerio* (NP_001017834), *Dimorphilus gyrociliatus* (CAD5123533), *Fistulifera solaris* (GAX13589), *Haliaeetus leucocephalus* (XP_010581340), *Homo sapiens* (NP_001338561), *Jimgerdemannia flammicorona* (RUP43481), *Klebsormidium nitens* (GAQ87748), *Latimeria chalumnae* (XP_005994715), *Limulus polyphemus* (XP_022236329), *Lipomyces starkeyi* (ODQ70434), *Mus musculus* (NP_036102), *Neurospora crassa* (CAD70389), *Penaeus monodon* (XP_037797824), *Petromyzon marinus* (XP_032824552), *Phaeodactylum tricornutum* (XP_002178064), *Raphidocelis subcapitata* (GBF90329), *Salpingoeca rosetta* (XP_004998634), *Selaginella moellendorffii* (XP_024528028), *Synchytrium microbalum* (XP_031023241), *T. lanuginosus* (this study and ANHP01000232), *Ustilago maydis* (XP_011387208), *Vitrella brassicaformis* (CEL97102) and *Xenopus laevis* (XP_018100075).

A multiple sequence alignment of the newly described *T. lanuginosus* polι protein and polι proteins from various other organisms was performed using the ClustalW algorithm in MacVector (version 15.5.4). Known polι protein sequences were obtained from Genbank protein records or identified by BLAST homology searches of Genbank genomic sequence records. Genbank accession numbers are indicated within the legend of Figure 1. The alignment was exported from MacVector in the Nexus sequence file format. This Nexus file was imported into the SplitsTree4 (version 4.14.4) and an unrooted phylogenetic tree was generated by setting the distance method to BioNJ.

### Purification of the *T. lanuginosus* DNA Polη

*E.coli* strain RW644 [F^*−*^ *dcm ompT hsdS*(rB^−^ mB^−^) *gal* λ(DE3) Δ*umuDC596*:*ermGT* Δ*dinB61*:*ble* Δ*araD-polB*:Ω Δ(*gpt-proA*)62] harboring pJM596 was grown overnight in 20 ml LB media containing 100 μg/ml ampicillin at 37°C. The overnight culture was then transferred into 1L fresh LB-ampicillin and grown at 37°C until an OD_600_ ∼0.5. At this point, IPTG was added to a final concentration of 1 mM to induce expression of *T. lanuginosus* DNA polη and grown for an additional 2 h before cells were harvested by centrifugation. The cell pellet was resuspended in 15 ml lysis buffer (50 mM Tris pH 8.0, 150 mM NaCl, 10 mM β-mercaptoethanol), sonicated, and cleared by ultracentrifugation at 45,000 rpm in a Beckman 50.2 Ti rotor for 45 min. Ammonium sulfate (45% saturation, 0.27 g/ml) was slowly added to the cleared lysate and stirred at 4°C for 30 min. Precipitated proteins were harvested by centrifugation and the resulting pellet resuspended in a buffer “A” (50 mM Tris pH 8.0, 20 mM NaCl, 10 mM β-mercaptoethanol, 20% v/v glycerol) and dialyzed overnight at 4°C against 1L of the same buffer. The dialyzed protein suspension was applied to a 5 ml HiTrap DEAE FF column (Cytiva, cat#17515401) and bound proteins were eluted with a 20–500 mM linear gradient of NaCl. Fractions containing polη were pooled and dialyzed against buffer “B” (10 mM sodium phosphate, pH 7.0,10 mM β-mercaptoethanol, 20% v/v glycerol) and applied to a 5 ml Bio-Scale™ Mini CHT™ Type II cartridge (BioRad, cat#7324332) and eluted with a 10–300 mM linear gradient of sodium phosphate in buffer B. Peak polη-containing fractions were pooled, aliquoted and stored at −80°C.

### Purification of the *T. lanuginosus* DNA Polκ-

*T. lanuginosus* DNA polκ was expressed in RW644 harboring pJM682 and purified using the same protocol as described above for polη.

### Purification of the *T. lanuginosus* DNA Polι

Our attempts to purify untagged *T. lanuginosus* polɩ using the above protocol for polη and polκ proved to be unsuccessful. We therefore decided to purify an N-terminal His-tagged version of *T. lanuginosus* polɩ. To do so, plasmid pJM966 was introduced into the *E.coli* SHuffle^®^ T7 Express strain [F’ *lac, pro, lacI* ^q^/Δ(*ara-leu*)*7697 araD139 fhuA2 lacZ*:T7 gene1 Δ(*phoA*)PvuII *phoR ahpC galE galK* λ*att*:pNEB3-r1-cDsbC (Spec^R^, *lacI* ^q^) *ΔtrxB rpsL150*(Str^R^) Δ*gor* Δ(m*alF*)3] (New England Biolabs, cat#C3029J). Expression, cell harvest, lysis, and clearance were performed as described above for polη and polκ and the cleared lysate was dialyzed overnight at 4°C against 1L of buffer “C” (20 mM Sodium Phosphate, pH 7.5, 500 mM NaCl, 20 mM imidazole, 10 mM β-mercaptoethanol, 10% v/v glycerol) The dialysed lysate was applied to a 5 ml HisTrap HP column (Cytiva, cat#17524701) and eluted with a linear 20 mM to 1 M imidazole gradient. Fractions containing polι were pooled and dialyzed against 1L of buffer “D” (10 mM Sodium Phosphate, pH 7.5, 300 mM NaCl, 10 mM β-mercaptoethanol, 20% v/v glycerol) and applied to a 5 ml Bio-Scale™ Mini CHT™ Type II cartridge (BioRad, cat#7324332) and eluted with a 10–300 mM linear gradient of sodium phosphate. Peak polι-containing fractions were pooled, aliquoted and stored at −80°C.

### Purification of *T. lanuginosus* Rev1

RW644 harboring pRARE2 (expressing tRNAs for seven rare codons; AGA, AGG, AUA, CUA, GGA, CCC, and CGG) was transformed with pJM863. The strain was grown overnight in 60 ml LB media containing 100 μg/ml ampicillin at 37°C. The overnight culture was then transferred into 3L fresh LB-ampicillin (1.5 L each in 2 x 4L flasks) and grown at 37 °C until an OD_600_ ∼0.5. At this point, IPTG was added to a final concentration of 1 mM to induce expression of *T. lanuginosus* Rev1 and grown for an additional 2 h before cells were harvested by centrifugation. The cell pellet was resuspended in 60 ml lysis buffer (50 mM Tris pH 7.5, 0.5 mM EDTA, 1 mM DTT, 20% glycerol), sonicated, and cleared by ultracentrifugation at 45,000 rpm in a Beckman 50.2 Ti rotor for 45 min. Ammonium sulfate (35% saturation, 0.2 g/ml) was slowly added to the cleared lysate and stirred at 4°C for 30 min. Precipitated proteins were harvested by centrifugation and the resulting pellet resuspended in a buffer “E” (10 mM Sodium phosphate pH 7.0, 25 mM NaCl) and dialyzed overnight against 2L of the same buffer at 4°C. The dialysed lysate was applied to a 20 ml HiPrep Heparin FF 16/10 column (Cytiva, cat#28936549) and eluted with a 25–400 mM NaCl linear gradient. Fractions containing Rev1 were pooled and applied to a HiPrep 16/60 Sephacryl S-200 HR size exclusion column (Cytiva, Cat#17116601) equilibrated in Phosphate Buffered Saline (PBS, pH6.8). Rev1 containing fractions were pooled and applied to a 5 ml Phosphocellulose P11 column (Whatman), equilibrated with buffer “F” (20 mM KPO_4_ pH 7.0, 100 μM EDTA, 10% glycerol, 1 mM DTT, and 100 mM KCl) and eluted with a 200–800 mM KCl linear gradient. Rev1-containing fractions were pooled, concentrated, and aliquoted for storage at −80°C.

### DNA Templates

Undamaged synthetic oligonucleotide primers and an 48 bp abasic site-containing template were synthesized by Lofstrand Laboratories (Gaithersburg, MD) using standard technique and PAGE purified. A synthetic abasic site, dSpacer, was purchased from Glen Research (Sterling, VA) and incorporated into the oligonucleotide template using standard techniques by Lofstrand Laboratories. The 7.2 kb M13mp18 circular template was purchased from New England Biolabs (Ipswich, MA). The *cis-syn* CPD containing oligonucleotide was synthesized by TriLink BioTechnologies (San Diego, CA) and has been described previously (Boudsocq et al., 2004). The synthesis of a template with BPDE-dA adduct was described previously (Frank et al., 2002). The sequence of each primer-template pair is given in the legend of the respective figures. Radiolabeled primers were labeled with [γ-^32^P]ATP using T4 polynucleotide kinase by Lofstrand Laboratories (Gaithersburg, MD). The fluorescent primer containing a 5′-Fluorescein (6-FAM), 5′-6FAM-ATGGTACGGACGTGCTT-3′, was synthesized by Lofstrand Laboratories. The template strand complementary to the fluorescent primer used in Figure 7B–D had the following sequence: 5′-ATT​AAC​GAA​TGAAG​CAC​GTC​CGT​ACC​ATC​G-3′, whereby the underlined base indicates the identity of the templating base. For Figure 7E, the sequences of the three template strands used to investigate damaged and undamaged bypass were also complementary to the fluorescent primer and are as follows: dG: 5′-ATT​AAC​GAA​TGAAG​CAC​GTC​CGT​ACC​ATC​G-3'; abasic site (X): 5′-ATTAACGAATXAAGCACGTCCGTACCATCG-3'; and CPD: 5′-TCG​ATA​CTG​GTA​CTA​ATG​ATT​AAC​GAATTAAG​CAC​GTC​CGT​ACC​ATC​G-3'. All primers were annealed to the unlabeled templates at a ratio of 1:2 by heating the primer and template together at 95°C in annealing buffer (0.1 M Tris-HCl, pH 8.0, 10 mg/ml BSA, 14.2 mM β-mercaptoethanol) and allowing the mixture to slowly cool to room temperature.

### Radiolabeled *in vitro* Primer Extension

Standard 10 μl reactions contained 50 mM Tris-HCl, pH 7.5, 100 μM each dNTP, 10 mM DTT, 10 nM primer-template DNA, unless specified otherwise in the legend, and supplemented with 4 mM MgCl_2_ for pols *η*- and *κ*-catalyzed reactions and 4 mM MnCl_2_ for polι-catalyzed reactions. Reactions were carried out at 37°C for 10 min except in thermostability and processivity experiments, where the temperature and duration varied as noted in the figure legends. Reactions were terminated by the addition of 10 μl of 95% Formamide, 17.5 mM EDTA, 0.025% Xylene cyanol, and 0.025% Bromophenol blue, heated at 95°C for 5 min, and briefly chilled on ice. Aliquots containing 5 μl of the samples were separated on 15% 8 M Urea polyacrylamide gels and visualized by PhosphorImager analysis.

### 6-FAM *in vitro* Primer Extension

For *in vitro* polymerase assays with *T. lanuginosus* Rev1, 12 μl reactions containing 50 mM Tris-HCl, pH 7.5, 10% glycerol w/v, 1 mM β-mercaptoethanol, 100 nM primer-template DNA, 5 nM *T. lanuginosus* Rev1, and 5 mM MgCl_2_ (with the exception of the Me^2+^ activation experiment) were initiated with 100 μM dNTP. Reactions were carried out at 37°C for 10 min except in the thermostability and processivity experiments, where the temperature and duration of experiments varied, respectively, as detailed in the figure legends. Reactions were terminated by the addition of 12 μl of 10 M Urea, 100 mM EDTA, 0.2% Xylene cyanol, and 0.02% Bromophenol blue, heated at 95°C for 5 min. Aliquots containing 12 μl of the samples were separated on 22% 8 M Urea polyacrylamide gels (Triple Wide Mini-Vertical gel system, C.B.S. Scientific) and visualized using 5′-fluorescein (6-FAM) fluorescence with a Typhoon FLA 7000 (GE Healthcare).

## Results and Discussion

### Identification of Y-Family Polymerases in *T. lanuginosus*

Based on previous investigations and observations, it has been noted that the genomes of higher eukaryotes, such as humans and mice, possess genes for the four known eukaryotic Y-family pols, whereas, the model organism *S. cerevisiae* only possesses genes for two of the eukaryotic Y-family pols, *RAD30* and *REV1*. Based on this observation, it has been suggested the genes encoding the closely related polη and polι proteins are paralogs of each other that diverged during evolution sometime after the origination of fungi. However, BLAST homology searches revealed that the genomes of filamentous fungi such as *Neurospora crassa* and *Aspergillus nidulans* and even the corn smut *Ustilago maydis* harbor a *POLI* gene, indicating to us that the genetic and biochemical characteristics of translesion synthesis in some “higher” fungi could be quite akin to that in higher eukaryotes such as humans. This notion of conserved functionality provided the impetus to embark on our efforts to identify and clone the four eukaryotic Y-family pols from the fungi, *T. lanuginosus*. Since *T. lanuginosus* is thermophilic, we reasoned the Y-family pols from this organism would exhibit enhanced stability during purification and biochemical analysis (see below).

In order to identify and clone the *T. lanuginosus* Y-family pols, protein sequence similarity comparisons were performed using known polη, polι, polκ, and Rev1 protein sequences, including other fungal sequences. Initially, degenerate PCR primers were designed based on highly conserved regions of protein homology between the various proteins within the four Y-family polymerase groups. Degenerate PCR was performed using *T. lanuginosus* genomic DNA and amplicons were cloned and sequenced. Subsequently, sequence of cloned regions of the Y-family polymerase genes were used to design gene-specific primers. A combination of various PCR techniques was then employed, for example degenerate PCR, nested PCR, RACE PCR, over-lapping exon PCR and flanking-sequence PCR using either genomic DNA or cDNA library DNA, to generate full-length sequences of each of the *T. lanuginosus POLH, POLI, POLK* and *REV1* genes. Full-length genes were then subcloned into protein expression vectors to facilitate purification of the four Y-family DNA pols. After we completed cloning the Y-family pol genes, the *T. lanuginosus* genome was sequenced and published allowing us to confirm that our cloned sequences do indeed encode the correct *T. lanuginosus* proteins. Sequence comparisons between *T. lanuginosus* and human Y-family pols reveal modest similarities that are higher than those observed between *S.cerevisiae* and human homologs. *T. lanuginosus* and human polη have 22% identical and 15% similar residues (compare to *S. cerevisiae* versus human at 16% identical and 15% similar residues). *T. lanuginosus* and human Rev1 have 24% identical and 15% similar residues (compare to *S. cerevisiae* versus human at 16% identical and 16% similar residues). Additionally, *T. lanuginosus* and human polκ have 20% identical and 14% similar residues, and *T. lanuginosus* and human polι have 24% identical and 16% similar residues.

### Phylogenetic analysis of the Y-Family Polymerase poli

The observation that the four eukaryotic Y-family pols are present in many filamentous fungi suggested to us that the divergence of *POLI* and *POLH* is even more ancient than the divergence of the fungi and metazoan (animals) groups. This supposition prompted us to perform a more in-depth phylogenetic analysis on the origins of the polι protein. We executed an exhaustive search for polι protein sequences within the Genbank database, including the fungi and metazoan groups, all other groups with clades containing various “lower” eukaryotes, and the plant kingdom. Although the *POLI* gene was absent from many “lower” or “simpler” eukaryotic organisms than fungi, as well as higher plants, we did find several examples of lower eukaryotes and simpler plants that do in fact encode a *POLI* gene.

A ClustalW alignment was performed using a group of sequenced *POLI* genes from organisms that include simple eukaryotes, lower plants, fungi and metazoans, all of which also possess genes for *POLH*, *POLK* and *REV1*. The unrooted phylogenetic tree of this alignment (Figure 1) reveals that the polι proteins cluster, for the most part, within several groups that include a chordate group, a lower animal grouping, including arthropods, annelids, echinoderms and mollusks, a fungi group, and a lower plant group. Polι proteins from other organisms that did not fit into these groups include a protein from a sea anemone (a Cnidarian), two proteins from diatom organisms and proteins from an Alveolate and a Choanoflagellate, which are examples of very simple single-celled eukaryotes. Interestingly, our analysis further supports the perception that the *POLI* gene is quite commonly found in fungal species, including some yeasts, and even other lower eukaryotic organisms and therefore substantiates our supposition that the evolutionary “split” between *POLH* and *POLI* may have preceded the emergence of fungi.

During this phylogenetic analysis of polι, one particularly noteworthy and unexpected observation was that *POLI* genes were quite frequently identified in some organisms within a particular group and absent in other organisms within that group. This finding holds true from the simplest eukaryotes to higher eukaryotes. For example, many protists do not possess a *POLI* gene such as Metamonads (*e.g.,* diplomonads, *i.e., Giardia lamblia* and parabasalids, *i.e., Trichomonas vaginalis*), Euglenozoans (*e.g.,* trypanosomatids, *i.e., Trypanosoma brucei* and *Leishmania donovani*) and Amoebozoans (*e.g., Entamoeba histolytica*). While other groups of protists do have a *POLI* gene such as Heteroloboseas (*e.g., Naegleria gruberi*) and Alveolates [*e.g., Vitrella brassicaformis* (Colpodellida clade)]. In direct contrast, other species of Alveolates in the Apicomplexans clade (*e.g., Cryptosporidium parvum*) and the Ciliophorans clade (ciliates) (*e.g., Paramecium tetraurelia*) do not have a *POLI* gene. In addition, some Stramenopiles have a *POLI* gene (*e.g.,* diatoms, *i.e., Phaeodactylum tricornutum* and *Fistulifera solaris*) and some do not (*e.g.,* diatoms, *i.e., Thalassiosira oceanica* and *e.g.,* water mould, *i.e., Saprolegnia parasitica*). Thus, it seems the presence, or absence, of a *POLI* gene in the genome of any specific organism is quite indiscriminate.

This unpredictable “hit or miss” trait of *POLI* holds true in plants, fungi, and metazoans as well. For example, we found *POLI* genes in the genomes of lower plant forms such as microalga, green algae, and spikemoss but were unable to find *POLI* genes in any higher vascular plants. A similar situation exists in fungi. No *POLI* genes were found in lower fungal forms such as Microsporidia and Glomeromycota. Likewise, in Saccharomycotina (true yeasts) (e.g., *S. cerevisiae*) no *POLI* genes were found. Some fungi in the Taphrinomycotina subphylum, such as *Saitoella complicate*, have *POLI* genes, while the closely related fission yeasts, *Schizosaccharomyces* species, do not. Furthermore, *POLI* genes were found in other yeast forms (*e.g.,* Lipomyces starkeyi), filamentous fungi, parasitic, and symbiotic fungi, and corn smut (Figure 1). However, we found no *POLI* genes in Basidiomycota which include club fungi and mushrooms.

Although most organisms within the various groups of animals, from the Choanoflagellates (considered to be the closest living relatives of the animals) up to the mammals, possess a *POLI* gene, we found it remarkable that there are numerous examples of higher eukaryotes organisms that do not possess a *POLI* gene. For example, we found no *POLI* genes in most Platyzoa (*i.e.,* Platyhelminthes, *e.g.,* flat worms), Rotifera (i.e., wheel animals), and Annelids (i.e., segmented worms), with the notable exception of *Dimorphilus gyrociliatus* which does harbor a *POLI* gene (Figure 1). While, many types of Mollusks do possess *POLI* genes, we found no *POLI* gene in octopi. Apparently, Arthopods such as spiders, scorpions, sea spiders, centipedes, millipedes and tardigrades (water bears) do not have *POLI*, but horseshoe crabs do. With a few exceptions, the majority of crustaceans and insects have *POLI*. Lastly, we found that the vast majority of chordates from the lancelet to humans do indeed possess a *POLI* gene (Figure 1). However, there are some extraordinary exceptions. We found that the tunicates (sea squirts), which are lower chordates, lack a *POLI* gene. Thus far, only a single fish species, *Esox lucius* (northern pike), was identified as lacking *POLI.* While alligators possess a *POLI* gene, the Australian saltwater crocodile (*Crocodylus porosus*) and the gharial (*Gavialis gangeticus*) both lack *POLI*. Most astoundingly, the majority of bird species, including Galliformes (*e.g.,* chickens, turkeys, grouse, quail, partridges and pheasants), Bucerotiformes (hornbills), Upupiformes (hoopoes) and the vast majority of Passeriformes (*e.g.,* sparrow, finch, tit, crow, canary, rifleman, and starlings) which includes more than half of all bird species, lack a *POLI* gene.

This phylogenetic analysis provides a “snapshot” of the evolution of *POLI* at this given moment in time. At some time in the past, the polη and polι paralogs diverged from one another due to a need to have two biochemically distinct translesion synthesis polymerases. The biochemical properties of both human polη and polι are well characterized. However, unlike polη which is defective in the human *Xeroderma Pigmentosum* Variant syndrome (Masutani et al., 1999), the cellular function of polι remains enigmatic (McIntyre, 2020; Vaisman and Woodgate, 2020). Therefore, the evolutionary pressure that led to this division of polη and polι remains a mystery. Perhaps this seemingly arbitrary “hit or miss” distribution of *POLI* genes in eukaryotic organisms can provide clues as to the cellular functioning of polι. Evaluation of the biological and biochemical translesion synthesis properties of polη and polι from closely related lower eukaryotes, one of which possesses a *POLI* gene and the other that does not, may provide hints as to when, and why, these two paralogs diverged and the cellular functions of polι.

### Expression and Purification of *T. lanuginosus* Polymerases η, ι, κ, and Rev1

**FIGURE 2:**
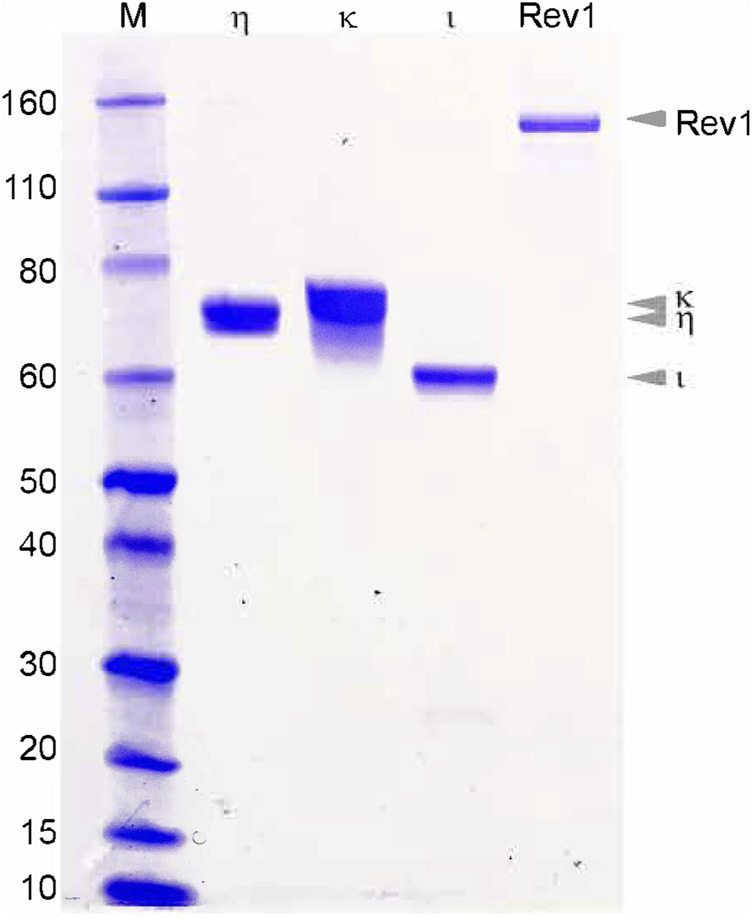
Purified *T. languinosus* polymerases η, κ, ι and Rev1. Two to five μg of the final purified polymerases were separated on a 12% SDS-PAGE gel. Visual analysis suggests each polymerase is greater than 95% pure.

All four Y-family pols from *T. lanuginosus* were expressed in *E. coli*, but their expression levels, solubility, and ease of purification varied. Both pols η and κ were well-expressed in *E. coli* and readily purified by conventional protocols including ammonium sulfate precipitation and ion-exchange chromatography (Figure 2). Rev1 was initially poorly expressed (unpublished observations), but expression increased dramatically in the presence of the pRARE2 plasmid coding for rare *E.coli* tRNAs. *T. lanuginosus* Rev1 was also purified by conventional methods including ammonium sulfate precipitation, size-exclusion and ion-exchange chromatography (Figure 2). *T. lanuginosus* polι was the most problematic of the four Y-family polymerases to purify, requiring codon optimization for *E.coli* expression and the addition of an N-terminal Histidine tag. Subsequently, the protein was purified by affinity- and ion exchange-chromatography (Figure 2). Once the polymerases were purified to >95% homogeneity, they were characterized for metal ion requirement, ability to bypass lesions, fidelity, thermostability, and processivity.

### Activation of *T. lanuginosus* Polymerases η, κ, ι by Mn^2+^ and Mg^2+^

DNA polymerases are known to require divalent cations to catalyze the nucleotidyl transfer reaction (Bessman et al., 1958; Mildvan and Loeb, 1979; Loeb and Kunkel, 1982; Vashishtha et al., 2016). Although it is generally believed that Mg^2+^ is the activating co-factor *in vivo* due to its cellular abundance, other divalent metal cations such as Mn^2+^, Co^2+^, Ni^2+^, Zn^2+^ have the ability to substitute for Mg^2+^ under certain conditions (Mildvan and Loeb, 1979; Loeb and Kunkel, 1982). Different metal co-factors can affect the activity and fidelity of DNA polymerases, promote TLS, and in the case of X-family enzymes, increase polymerization efficiency (Martin et al., 2013). In rare cases, Mn^2+^ appears to serve as a natural metal activator of DNA polymerases. This has been shown for X-family polymerases λ and μ and Y-family polι which exhibit great preference for Mn^2+^ over Mg^2+^ as the activating metal ion (Dominguez et al., 2000; Frank and Woodgate, 2007; Garcia-Diaz et al., 2007). In particular, human polι was inhibited by MgCl_2_ present at physiological concentrations and exhibited peak activity at low MnCl_2_ levels (0.05–0.25 mM). Furthermore, at optimal concentrations, Mn^2+^ ions improved the fidelity of polι-catalyzed nucleotide incorporation opposite template dT.

**FIGURE 3:**
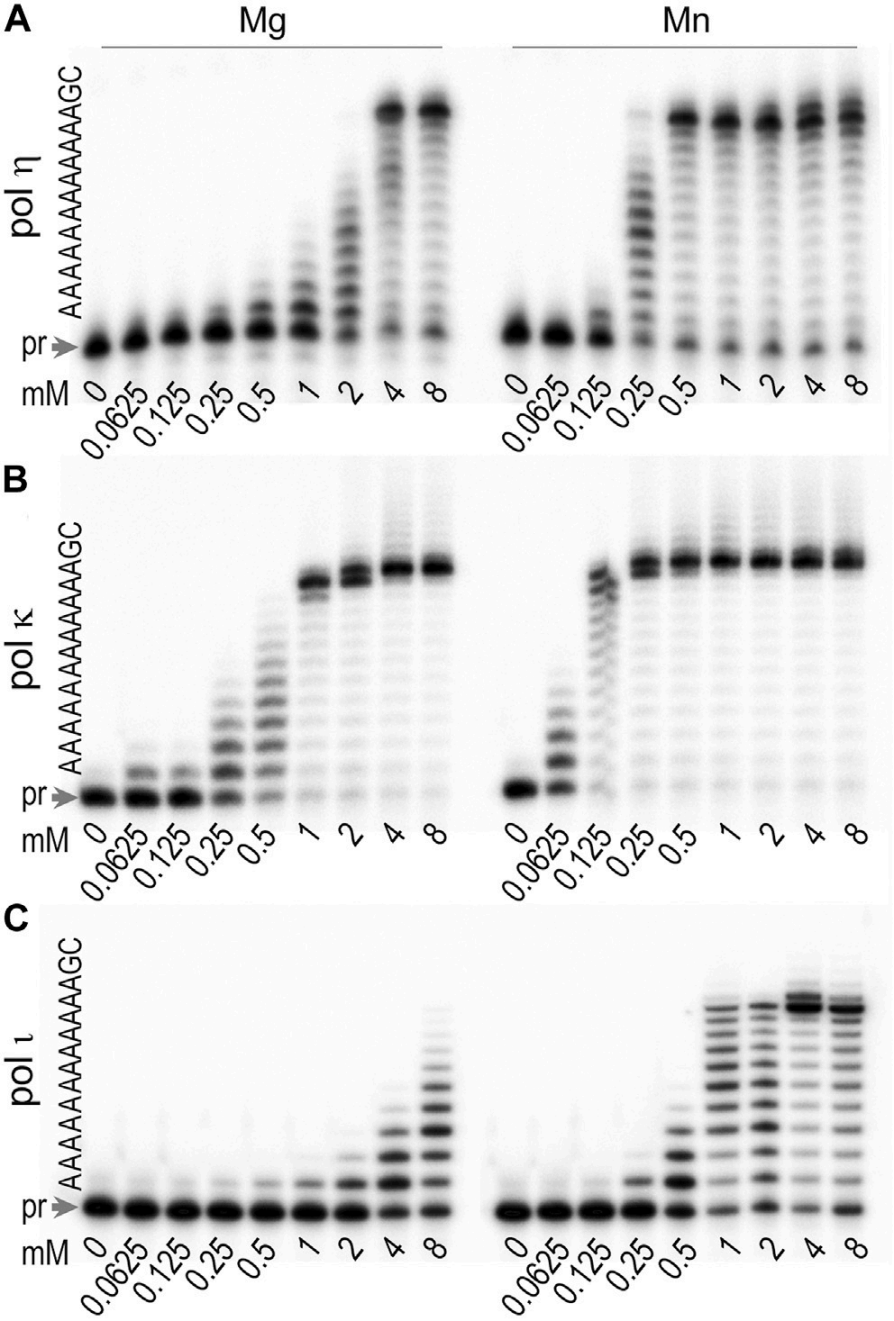
Determination of metal ion requirements (type and concentration) for optimal activity of **(A)** polη **(B)** polκ, and **(C)** polι. The extension of ^32^P-labeled primer 5′-CGA​TGG​TAC​GGA​CGT​GCT​T-3′ hybridized to a template 3′-GCT​ACC​ATG​CCT​GCA​CGA​AAA​AAA​AAA​AAG​C-5′ was carried out for 10 min at 37°C in the presence of 100 μM dNTPs mixture and with increasing concentrations of MgCl_2_ or MnCl_2,_ ranging from 0.0625 to 8 mM. Concentrations of enzymes were 0.17 pM for polη, 0.32 pM for polκ, and 0.15 pM for polι. The sequence of the template immediately downstream of the primer (pr) is shown on the left-hand side of each gel pair.

We were interested in determining the optimal concentration and type of divalent metal ion that is required for activation of *T. lanuginosus* pols η, κ, and ι. We assessed the primer extension activity of these polymerases in the presence of Mg^2+^ and Mn^2+^ at concentrations ranging from 62.5 μM to 8 mM (Figure 3). Polη exhibited greatest activity in reactions with 4–8 mM Mg^2+^ and 0.5–8 mM Mn^2+^ (Figure 3A). Reactions catalyzed by polκ were most efficient at 1–8 mM Mg^2+^ and 0.25–8 mM Mn^2+^ (Figure 3C). Polι was most active in the presence of ∼4 mM Mn^2+^ (Figure 3B).

As shown in Figure 3, Mn^2+^ appears to be a more conducive co-factor than Mg^2+^ in reactions with all three *T. lanuginosus* pols, as they all were active over a wider range of Mn^2+^ and exhibited greater activity at the same concentration of Mn^2+^ vs. Mg^2+^. However, there are some interesting differences seen between the reported metal ion requirements for human and *T. lanuginosus* polι. Human polι exhibits highest activity at 0.05–0.2 mM MnCl_2_ (Frank and Woodgate, 2007), whereas *T. lanuginosus* enzyme required much higher concentrations of Mn^2+^ with a sharp peak of activity at ∼4 mM MnCl_2_. In contrast, while human polι was inhibited by MgCl_2_ at concentrations >1 mM, *T. lanuginosus* polι required at least 0.5 mM Mg^2+^ and its activity gradually increased with concentrations up to 8 mM.

Because Mn^2+^ is generally known to decrease replication fidelity, we used 4 mM Mg^2+^ for the subsequent studies with polη and polκ. In contrast, further characterization of polι was performed in the presence of 4 mM Mn^2+^.

### Processivity of *T. lanuginosus* Polymerases η, ι and κ

**FIGURE 4:**
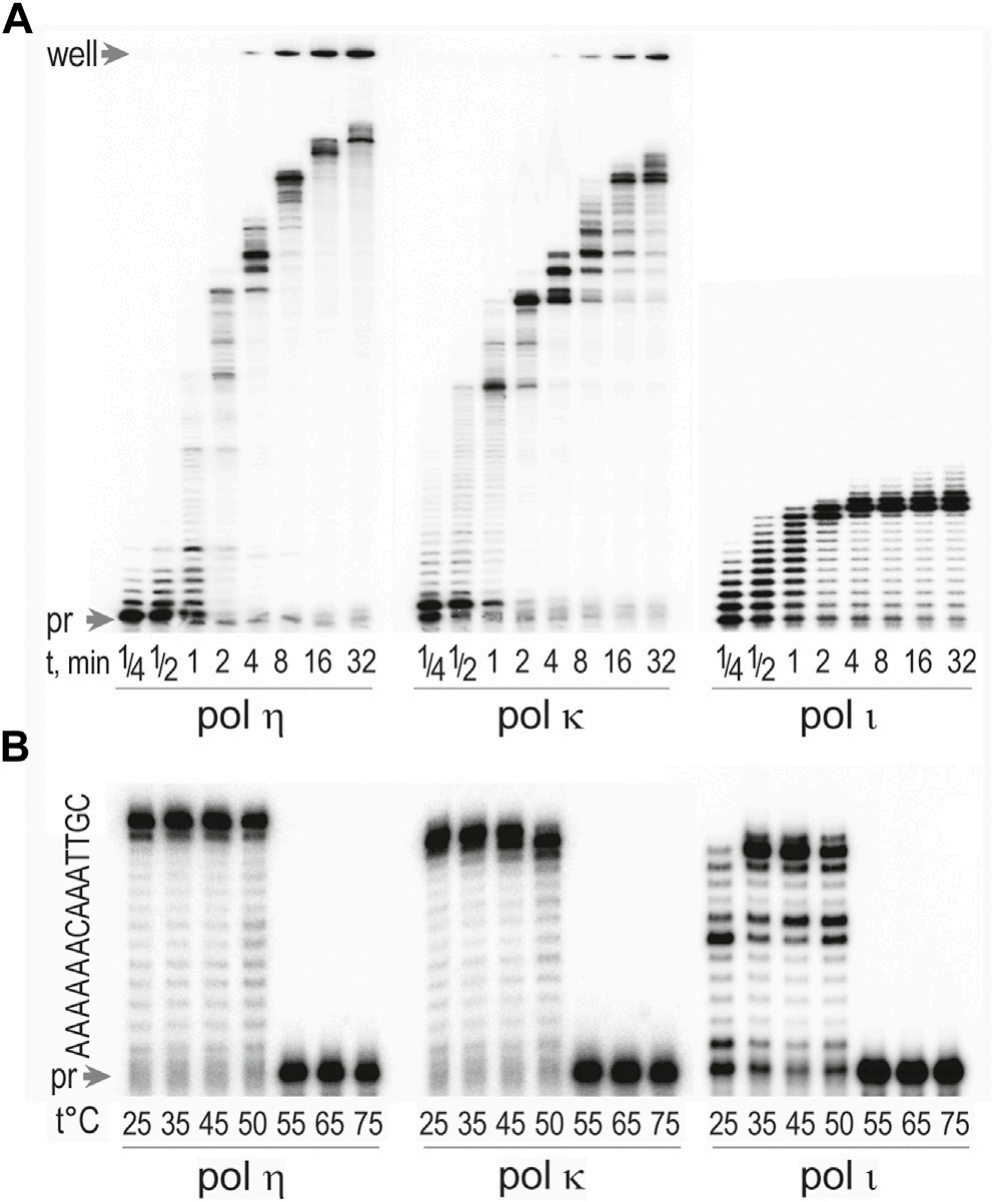
Thermostability and processivity of *Thermomyces lanuginosus* pols η, κ, and *ι*. Concentrations of enzymes were 0.17 pM for pol η, 0.32 pM for pol κ, and 0.15 pM for pol ι. **(A)** Processivity of pols η, κ, and ι were assayed in reactions containing 100 μM dNTPs and 4 mM MnCl_2_ for polι or MgCl_2_ for polη and polκ. Extension of ^32^P labeled primer 5′ TAT​TTA​TCC​CAA​TCC​AAA​TAA​GAA​ACG​A-3′ hybridized to the circular single-stranded 7.2 kb M13mp18 bacteriophage was at 37°C for various times ranging from 0.25 to 32 min. Reactions containing 100 μM dNTPs and 4 mM MnCl_2_ for reactions with polι and MgCl_2_ for reactions with polη and polκ. (**B**) Thermostability was assayed by primer extension reactions using DNA template generated by annealing of a^32^P-labeled primer 5′-TAT​TTA​TCC​CAA​TCC​AAA​TAA​GAA​ACG​A-3′ with an undamaged DNA template 5′-CGT​TAA​ACA​AAA​AAT​CGT​TTC​TTA​TTT​GGA​TTG​GGA​TAA​ATA-3'. Reactions were carried out for 10 min at various temperatures ranging from 25 to 75°C and contained 100 μM dNTPs and 4 mM Mn^2+^ to activate polι or Mg^2+^ to activate polη and polκ.

We next examined the processivity of *T. lanuginosus* pols η, ι, and κ (Figure 4A). As with the human enzymes, *T. lanuginosus* polη and polκ were much more processive than polι and incorporated ∼6 and ∼35 bases, respectively, in a single binding event after only 1 min. In contrast, polι incorporated ∼10–11 bases in a distributive manner. It then exhibited a strong pause after encountering two adjacent template dTs located 11 and 12 nucleotides from the 5′ end of the primer. Presumably this is due to misincorproration of dG opposite the dTs and its subsequent poor extension (Vaisman et al., 2001). These results indicate that the processivity of *T. lanuginosus* pols η, ι, and κ are therefore similar to their human counterparts (Washington et al., 1999; Ohashi et al., 2000a; Tissier et al., 2000b).

### Thermostability of *T. lanuginosus* Polymerases

*T. lanuginosus* is a thermophilic fungus with a natural habitat of growing in organic soils, *e.g.,* decomposing vegetable matter, composts, and animal excrements, at an optimal temperature between 48 and 52°C (Tsiklinsky, 1899; Maheshwari et al., 2000; Singh et al., 2003).The ability of this fungus to live in such an environment suggests that its proteins possess enhanced thermostability (Maheshwari et al., 2000). We therefore examined the optimum temperature for the *T. lanuginosus* pols η, κ, and ι by incubating primer extension reactions at temperatures ranging between 25 and 75°C. All three polymerases exhibited robust activity between 25 and 50°C, but there was sharp decline in activity at temperatures greater than 50°C (Figure 4B).

### Polη, Polι, and polκ-dependent Lesion Bypass Past Abasic Sites, CPDs, and benzo[*a*] Pyrene Adducts

The Y-family polymerases are best characterized by their ability to catalyze translesion replication past a variety of DNA lesions, in a manner distinct from the cell’s conventional replicases (Sale et al., 2012; Vaisman and Woodgate, 2017). We were therefore interested in assessing the ability and fidelity of *T. lanuginosus* pols η, κ, ι and to carry out translesion synthesis across a *cis-syn* CPD, an abasic site, and a benzo[*a*]pyrene adduct.

Apurinic/apyrimidinic (or abasic) sites are generated as a result of spontaneous or enzymatically-induced base loss and are considered the most frequent form of DNA damage (Lindahl, 1993; Boiteux and Guillet, 2004). An estimated 10,000 abasic sites occur per human cell per day under physiological conditions (Loeb, 1989). It is therefore not surprising that the ability of polη to copy DNA templates containing this noninstructional lesion has been extensively investigated. What is surprising, is that studies performed by various groups with human and *S. cerevisiae* polη produced conflicting results, ranging from those stating that “hPolη has the highest abasic lesion bypass efficiency” among human Y-family DNA pols and “is a major pol involved in abasic site bypass” (Choi et al., 2010; Sherrer et al., 2011; Patra et al., 2015; Thompson and Cortez, 2020) to those which dismiss human, or *S. cerevisiae* polη, as contributing to *in vivo* abasic site bypass because of “negligible nucleotide insertion opposite the abasic site and primer extension past the lesion” (Haracska et al., 2001; Pages et al., 2008). Somewhere in between these polar extremes are studies suggesting that polη catalyzes insertion and/or extension steps of abasic site bypass efficiently enough to ensure its involvement in TLS *in vivo*, but not as a major player (Zhang et al., 2000a; Masutani et al., 2000; Zhao et al., 2004; Sherrer et al., 2011).

**FIGURE 5:**
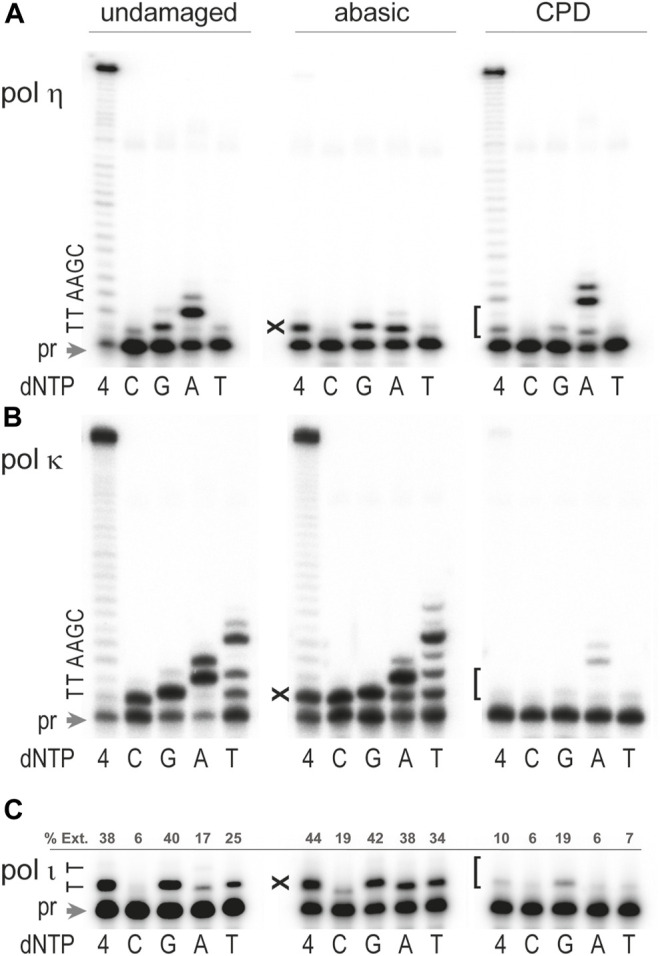
Bypass of abasic and CPD site by *Thermomyces lanuginosus* polymerases. The ability to bypass abasic site and CPD by **(A)** polη, **(B)** polκ, and **(C)** polι was assayed by measuring extension of ^32^P labeled primer 5′-CGA​TGG​TAC​GGA​CGT​GCT​T-3′ hybridized to the undamaged or damaged DNA template. 5′-TCGATACTGGTACTAATGATTAACGAA- **TN** -AAGCACGTCCGTACCATCG-3'. The underlined bold TN sequence stands for the undamaged TT, TT-CPD, or TX, where X is an abasic site, pr is unextended primer. Reactions contained 100 μM each individual nucleotide (dC, dG, dA, dT) or mixture of all four dNTPs as indicated in the Figure, and 4 mM Mn^2+^ for reactions catalyzed by polι or Mg^2+^ for reactions with polη and polκ. Primer extension reactions were carried out at 37°C for 10 min. Concentrations of enzymes were 0.17 pM for pol η, 0.32 pM for polκ, and 0.15 pM for polι. The sequence of the template immediately downstream of the primer (pr) is shown on the left-hand side of each gel pair. “X” denotes the position of the abasic site and “[” indicates position of the T-T CPD. For panel C, primer extension (%Ext.) was quantified using ImageJ software and reported as the percentage of product density relative to total density for each reaction.

We show here that the efficiency and fidelity of *T. lanuginosus* polη during TLS of abasic sites is reminiscent of properties of human and yeast polη uncovered in the third group of studies described above (Zhang et al., 2000a; Masutani et al., 2000; Zhao et al., 2004). In particular, in standing-start primer extensions *T. lanuginosus* polη inserted dA and dG opposite the abasic site with moderate efficiency, but further primer elongation was largely obstructed (Figure 5A). However, minuscule extension of the primer with terminal dA opposite the abasic site occurred by incorporation of an additional dA when the base 5′ to the abasic site was dT (Figure 5A, incorporation of dA). It should be noted that base selectivity of polη on the template with an abasic site is similar to its moderate fidelity on the undamaged DNA. As shown in Figure 5A, polη preferentially incorporates a correct dA opposite the undamaged dT although substantial misinsertion of dG and low level of dC and dT misincorporation can also be seen.

As with polη, there is no consensus regarding the ability of the DinB orthologs (polκ) to utilize DNA templates containing abasic sites (Strauss, 1991; Ohashi et al., 2000b; Zhang et al., 2000b; Boudsocq et al., 2001; Gerlach et al., 2001; Choi et al., 2010; Sherrer et al., 2011), which is at least partly explained by the strong effect of the surrounding sequence context on the bypass mechanism, base selectivity, and efficiency of insertion and extension steps of TLS. Thus, it has been shown that human polκ tolerates an abasic site much better when the next (5′) template base is dT, and the nucleotide preferentially incorporated opposite the lesion is dA. Furthermore, when primer/template slippage is possible, bypass often occurs through a stabilized misalignment mechanism leading to 1– or 2-base deletions (Boudsocq et al., 2001; Ling et al., 2004a). Similarly, *T. lanuginosus* polκ-catalyzed TLS of the abasic site was quite efficient when the 5′ template was dT and dA was preferentially incorporated opposite the lesion, although the other three nucleotides were also inserted at substantial levels (Figure 5B). The low fidelity of polκ during abasic site bypass is very similar to its highly error-prone nucleotide incorporation on undamaged DNA. The fact that the major product of *T. lanuginosus* polκ primer extension reactions in the presence of dT was generated by addition of four nucleotides is consistent with the aforementioned human polκ template-slippage mechanism (Mukherjee et al., 2013). Therefore, the resulting main product would contain two mismatches and total primer elongation by four bases because the DNA template used in the current study has two dA’s next to dT. When the DNA template was not susceptible to primer/template slippage (reactions with dC, dG, and dA) only one wrong nucleotide was incorporated opposite the abasic site.

Human polι has the capacity to insert deoxynucleotides opposite an abasic site, however further extension is limited (Zhang et al., 2000c; Vaisman et al., 2002; Nair et al., 2009; Choi et al., 2010; Sherrer et al., 2011). *T. lanuginosus* polι behaved in a similar manner, *i.e.,* a single nucleotide was readily inserted opposite the abasic site with no indication of further extension (Figure 5C). Furthermore, akin to human polι, *T. lanuginosus* polι incorporates dG, dA, and dT with similar efficiency. Interestingly, when undamaged templates were used, *T. lanuginosus* polι was even more inaccurate than the human polymerase, *i.e.,* not only dG, but also dT, was inserted opposite template dT more often than the correct dA (Figure 5C).

The most common ultraviolet light photoproduct is a *cis-syn* cyclobutane pyrimidine dimer (CPD). Unlike the ambiguity of TLS of an abasic site, there is no question as to which TLS polymerase bypasses a CPD most efficiently. Indeed, it is well established that polη can bypass a CPD with the same efficiency and accuracy as undamaged template dT’s (Johnson et al., 2000c; Masutani et al., 2000; McCulloch et al., 2004). Most other pols halt synthesis either immediately before the lesion, or opposite the first template dT of the CPD, as their active site is unable to accommodate the covalently joined dT’s of the CPD. Similar to human and *S. cerevisiae* polη, *T. lanuginosus* polη efficiently bypassed the CPD lesion (Figure 5A). Like polymerases purified from other species, *T. lanuginosus* polη was even more accurate while inserting nucleotides opposite the damaged bases than on undamaged DNA templates (Figure 5A).

The pols in the DinB branch of the Y-family (polκ) are unable to replicate past UV-induced *cis-syn* CPD dimers (Johnson et al., 2000a; Ohashi et al., 2000b; Zhang et al., 2000b) with the one reported exception of archaeal *Sulfolobus solfataricus* Dpo4, whose properties resemble eukaryotic polη (Boudsocq et al., 2001). Interestingly, the ability of *T. lanuginosus* polκ to copy a CPD-containing template resembles the behavior of its human orthologs rather than the thermophilic archaeal enzyme, *i.e., T. lanuginosus* polκ was virtually blocked by the CPD lesion in reactions with all four dNTPs although a very low level of the correct incorporation of two dAs opposite the damaged bases was detected in reactions where only dATP was present (Figure 5B).

It has been reported that human polι can bypass a CPD. Depending on the sequence context, it incorporates anywhere between 1–5 nucleotides, albeit with low efficiency (Tissier et al., 2000a; Vaisman et al., 2003). *T*. *lanuginosus* polι was much less efficient compared to the human enzyme. In fact, CPD bypass was barely detectible (Figure 5C). As with undamaged template dT, the preferred nucleotide inserted opposite the 3′-dT of the CPD was dG (Figure 5C).

Benzopyrene diol epoxides (BPDEs) are the highly carcinogenic metabolites of the environmental pollutant benzo[*a*]pyrene, found in tobacco smoke and automobile exhaust. In mammalian cells, various BPDE stereoisomers covalently bind to DNA forming adducts at the N^2^ and N^6^ position of guanine and adenine, respectively. Previous studies demonstrated that efficiency and fidelity of TLS past BPDE by different pols not only depends on the DNA base to which the adduct is linked, but is also often modulated by the stereochemistry of the adduct (Zhang et al., 2002a; Chiapperino et al., 2002; Frank et al., 2002; Rechkoblit et al., 2002; Suzuki et al., 2002; Jia et al., 2008). For example, in general, human polκ is almost completely stalled by BPDE-dA adducts. Under similar reaction conditions, it accurately and efficiently bypasses most of the BPDE-dG stereoisomers (Zhang et al., 2002a; Rechkoblit et al., 2002; Suzuki et al., 2002; Jia et al., 2008), although the *cis-S*-BPDE-dG represents a significant block for the polymerase (Suzuki et al., 2002). In contrast, polι is much more efficient and accurate while incorporating nucleotides opposite BPDE-dA than opposite BPDE-dG (Frank et al., 2002). Furthermore, independent of adduct stereochemistry, the correct dT is inserted by polι equally efficiently opposite various BPDE-dA isomers. On the other hand, the ability of human polη to replicate past the BPDE is strongly affected by the adduct conformation and damaged base, *i.e.,* it readily traverses through the *trans-S*-BPDE-dA, but not through the isomeric *trans-R-* adduct, or through the BPDE-dG isomers (Chiapperino et al., 2002; Rechkoblit et al., 2002). In the current study with *T. lanuginosus* pols, we used DNA templates with a *trans-S*-BPDE-dA adduct. Reactions with polη and polκ contained Mg^2+^ or Mn^2+^, whereas polι was tested only in the presence of Mn^2+^.

**FIGURE 6:**
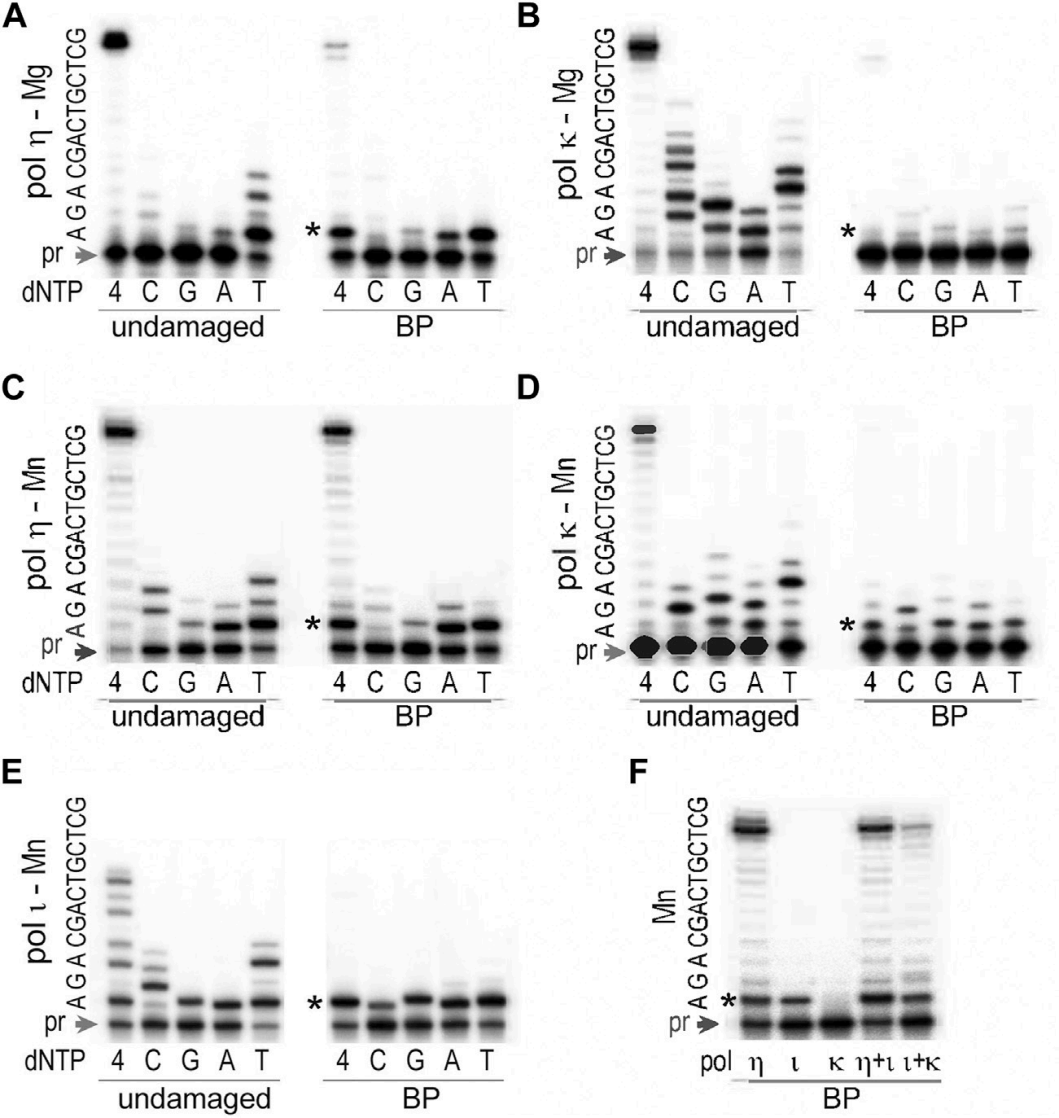
TLS past *trans*-*S*-BPDE-dA by *T. lanuginosus* pols. The ability to bypass BPDE-dA was assayed for **(A)** polη in presence of 4 mM Mg^2+^, **(B)** polκ in presence of 4 mM Mg^2+^, **(C)** polη in presence of 4 mM Mn^2+^, **(D)** polκ in presence of 4 mM Mn^2+^, **(E)** polι in presence of 4 mM Mn^2+^, and **(F)** individual, or mixture of various pols in 4 mM Mn^2+^. The substrate used in these assays was made by annealing of the ^32^P labeled primer 5′-CAC​TGC​AGA​CTC​TAA​A-3′ and either an undamaged or BPDE-containing template 5′- GCT​CGT​CAG​CAG **A** TTT​AGA​GTC​TGC​AGT​G-3′, where the underlined bold A stands for the undamaged, or BPDE-modified dA. Reactions contained 100 μM each individual nucleotide (dC, dG, dA, dT) or mixture of all four dNTPs as indicated in the figure and were carried out at 37°C for 10 min. Concentrations of enzymes were 0.17 pM for polη, 0.32 pM for polκ, and 0.15 pM for polι. The sequence of the template immediately downstream of the primer (pr) is shown on the left-hand side of each gel pair. The star (*) indicates position of the adduct.

Similar to the human enzyme, *T*. *lanuginosus* polη was quite efficient, but rather inaccurate, while incorporating nucleotides opposite the *trans-S*-BPDE-dA (Figures 6A,C) especially when reactions were carried out in the presence of Mn^2+^ which significantly boosted TLS efficiency without noticeably reducing fidelity (Figure 6C). Nucleotide incorporation opposite the lesion by polι was at least as efficient as by polη (Figure 6E), but it was less accurate and further primer extension was inhibited (Figure 6E). We have previously found that fidelity of human polι was influenced by the local sequence context of the BPDE-dA lesion (Frank et al., 2002), and when the template bases 5′ to the lesion were GAC, uncharacteristically accurate insertion of dT opposite the BPDE-dA was observed. In contrast, it appears that on the same template, *T. lanuginosus* polι keeps its general unfaithful nature. Finally, *T*. *lanuginosus* polκ when activated by Mg^2+^ barely showed any activity on the BPDE-dA-adducted DNA (Figure 6B). However, when Mg^2+^ was substituted by an equimolar concentration of Mn^2+^ (Figure 6D), *T. lanuginosus* polκ was able to utilize this template adding 1–2 nucleotides to the primer. Incorporation of all four nucleotides in the presence of 4 mM Mn^2+^occurred with similar efficiency, making synthesis by polκ highly error-prone (Figure 6D).

While polκ itself was incapable of extending primers by more than one nucleotide after insertion opposite the BPDE-dA even in the presence of Mn^2+^, it was able to assist polι to bypass the lesion and elongate primers to the end of the template (Figure 6F). Nevertheless, TLS catalyzed by the combined action of polι and polκ was still less efficient than by polη alone (Figure 6F).

### Characterization of *T. lanuginosus* Rev1

**FIGURE 7:**
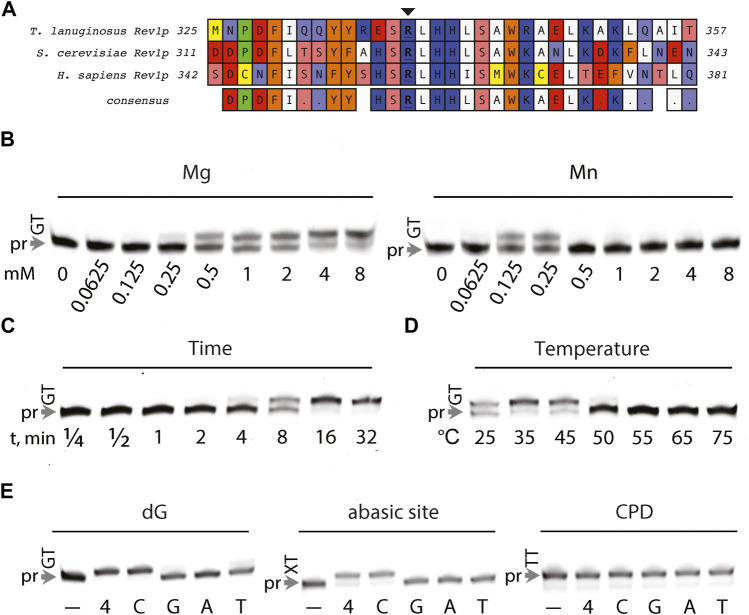
Characterization of DNA synthesis by *T. lanuginosis* Rev1. **(A)** Sequence alignment between REV1 orthologs (*Thermomyces lanuginosus, Saccharomyces cerevisiae, and Homo sapiens*), illustrating the conserved arginine residue (indicated with an arrow) required for protein-templated DNA synthesis **(B)** Serial 1:1 dilutions of Mg^2+^ and Mn^2+^ metal ions present within 6-FAM primer extension reactions, starting at 8 mM Me^2+^. **(C)** Processivity was qualitatively assessed by varying the duration (minutes) of 6-FAM primer extension reactions. **(D)** Thermostability was evaluated by monitoring DNA synthesis activity at varied temperature (25–75°C). **(E)** Lesion bypass activity for undamaged (dG), abasic site, and CPD template base/lesion identities was examined in the presence of either none (–), 100 µM dNTPs (4), 100 µM dCTP (C), 100 µM dGTP (G), 100 µM dATP (A), 100 µM dTTP (T).

*S. cerevisiae* and human Rev1 function as a dCMP transferase, incorporating strictly dCMP opposite abasic sites and dG-lesions (Nelson et al., 1996; Lin et al., 1999; Masuda et al., 2001). dCMP transferase activity is instructed by Rev1’s conserved active site side chain arginine residue *via* a protein-templated mechanism (Nair et al., 2005; Weaver et al., 2020). We hypothesized that the conservation of the functional arginine and Rev1 consensus sequence (Figure 7A) would make *T. lanuginosis* Rev1 a bonafide dCMP transferase. Therefore, the DNA synthesis activity of *T*. *lanuginosus* Rev1 was characterized *in vitro* opposite damaged and undamaged DNA. We found that *T. lanuginosus* Rev1 could utilize both Mn^2+^ and Mg^2+^, but reasoned that Mg^2+^ was a more likely candidate for physiological utilization given its wider efficacy in comparison to Mn^2+^ (Figure 7B). Similar to the activities of *S. cerevisiae* and human Rev1, DNA synthesis by *T*. *lanuginosus* Rev1 was not processive, whereby it inserted only once opposite a templating dG and stalled at the following dT base (Figure 7C). As expected, *T*. *lanuginosus* Rev1 had a similar thermostability to the other *T*. *lanuginosus* Y-family pols (Figure 7D). The well-characterized dCMP transferase activity of Rev1 ortholog was evaluated opposite undamaged, as well as abasic site- and CPD-containing templates (Figure 7E). The ability for *T*. *lanuginosus* Rev1 to perform DNA synthesis was efficient only in the presence of dCMP opposite undamaged, or abasic site templates, akin to *S. cerevisiae* and human REV1 (Zhang et al., 2002b; Nair et al., 2011; Weerasooriya et al., 2014). In summary, we found that the DNA synthesis properties of *T*. *lanuginosus* Rev1 are comparable with its higher eukaryotic ortholog.

## Concluding Remarks

In this paper, we describe the cloning, purification, and biochemical characterization of Y-family pols from the thermophilic fungus *T. lanuginosus. T. lanuginosis* contains all four eukaryotic Y-family orthologs that each generally demonstrate biochemical properties similar to the properties of higher eukaryotic orthologs with the exception of the expected thermostability of the *T. lanuginosis* Y-family polymerases. Interestingly, while *T. lanuginosis* polι exhibited biochemical properties akin to the human enzyme, its metal ion requirement was significantly different. Whereas human polι is activated by very low levels of Mn^2+^ (250 μM) and inhibited by high levels of Mg^2+^ (>1 mM) (Frank and Woodgate, 2007), *T. lanuginosis* polι required 4–8 mM Mn^2+^ or Mg^2+^ respectively, for maximal activity *in vitro*.

The conservation of Y-family polymerase activities between lower and higher eukaryotic organisms suggests that TLS activity may be more essential to cellular viability than previously appreciated. Further structure-function studies of these polymerases, as well as identification and characterization of other Y-family orthologs from yet unsequenced species would help clarify the seemingly random ortholog dispersion among eukaryotes and how their presence/absence might provide evolutionary advantages in the face of different environmental stressors.

## Data Availability

The raw data supporting the conclusions of this article will be made available by the authors, without undue reservation.

## Abbreviations

Pols, polymerases; TLS, translesion synthesis; CPD, cyclobutane pyrimidine dimer; BPDE, benzo[*a*] pyrene diol epoxide; *S. cerevisiae*, *Saccharomyces cerevisiae*; *T. lanuginosus*, *Thermomyces lanuginosus*; *S. pombe*, *Schizosaccharomyces pombe*.
