# Corrigendum: Identification and Characterization of Thermostable Y-Family DNA Polymerases η, ι, κ and Rev1 From a Lower Eukaryote, *Thermomyces lanuginosus*

**DOI:** 10.3389/fmolb.2021.819157

**Published:** 2021-12-15

**Authors:** Alexandra Vaisman, John P. McDonald, Mallory R. Smith, Sender L. Aspelund, Thomas C. Evans, Roger Woodgate

**Affiliations:** ^1^ Laboratory of Genomic Integrity, National Institute of Child Health and Human Development, National Institutes of Health, 9800 Medical Center Drive, Bethesda, MD, United States; ^2^ New England Biolabs Incorporated, Ipswich, MA, United States

**Keywords:** thermostable fungi, Y-family DNA polymerases, phylogenetic analysis, translesion DNA synthesis, DNA polymerase η, DNA polymerase ι, DNA polymerase κ, REV1

In the original article, there was a formatting issue in Figure 6 as published. This occurred when the image was converted from a PC generated pdf to an Apple Macintosh generated tif for publication. The corrected Figure 6 appears below.

**FIGURE 6:**
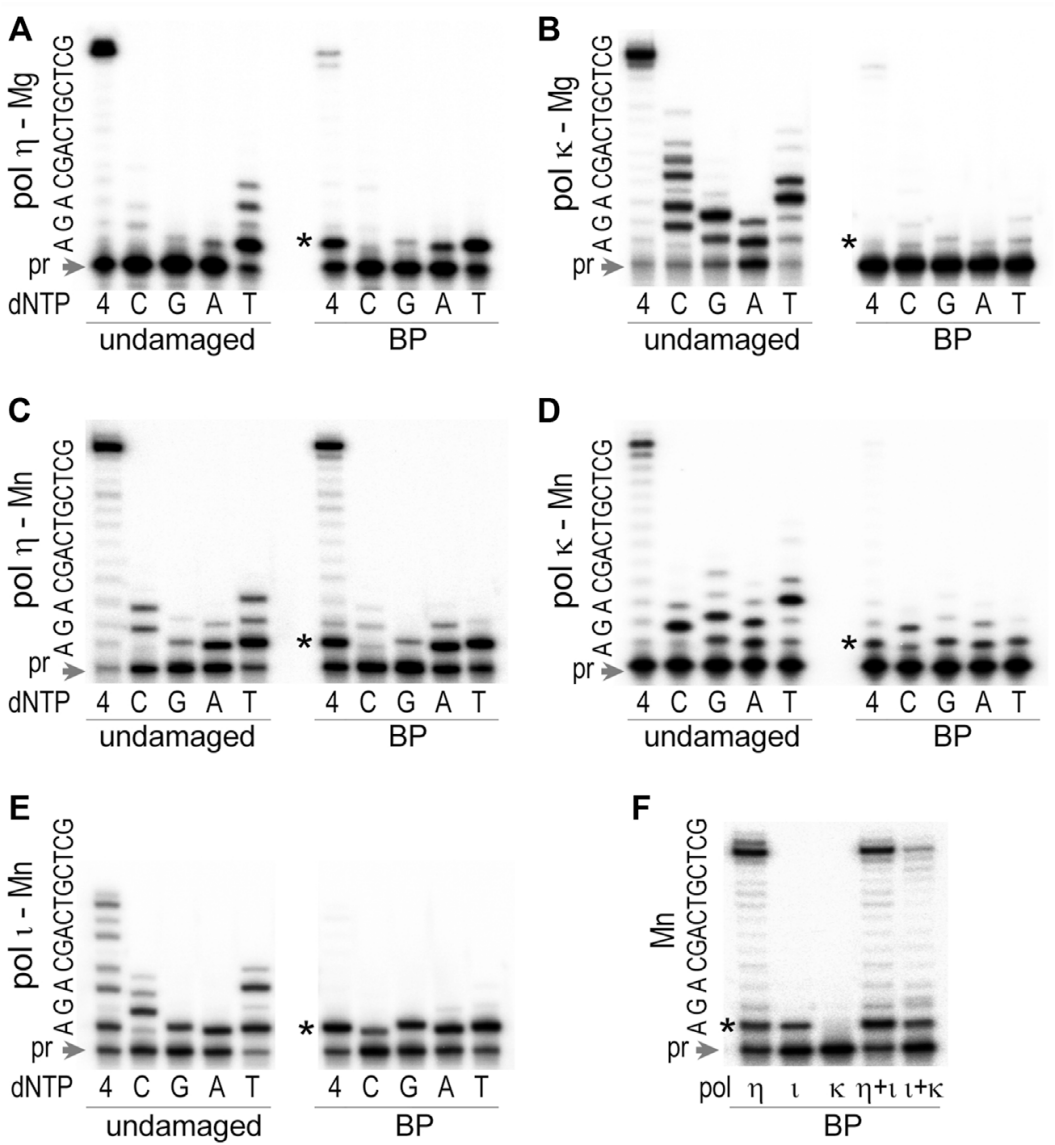
TLS past *trans-S*-BPDE-dA by *T. lanuginosus* pols. The ability to bypass BPDE-dA was assayed for **(A)** polη in thepresence of 4 mM Mg^2+^, **(B)** polκ in the presence of 4 mM Mg^2+^, **(C)** polη in the presence of 4 mM Mn^2+^, **(D)** polκ in the presence of 4 mM Mn^2+^, **(E)** polι in the presence of 4 mM Mn^2+^, and **(F)** individual, or a mixture of various pols in 4 mM Mn^2+^. The substrate used in these assays was made by annealing of the ^32^P labeled primer 5′-CAC​TGC​AGA​CTC​TAA​A-3′ and either an undamaged or BPDE-containing template 5′- GCT​CGT​CAG​CAG​ **A** TT​TAG​AGT​CTG​CAG​TG-3′, where the underlined bold A stands for the undamaged, or BPDE modified dA. Reactions contained 100 μM each of individual nucleotide (dC, dG, dA, and dT) or a mixture of all four dNTPs as indicated in the figure and were carried out at 37°C for 10 min. Concentrations of enzymes were 0.17 pM for polη, 0.32 pM for polκ, and 0.15 pM for polι. The sequence of the template immediately downstream of the primer (pr) is shown on the left-hand side of each gel pair. The star (*) indicates the position of the adduct.

The authors apologize for this error and state that this does not change the scientific conclusions of the article in any way. The original article has been updated.

